# Use of deep learning to segment bolus during videofluoroscopic swallow studies

**DOI:** 10.1088/2057-1976/ad0bb3

**Published:** 2023-11-23

**Authors:** Nadeem Shaheen, Ryan Burdick, Rodolfo Peña-Chávez, Christopher Ulmschneider, Joanne Yee, Atsuko Kurosu, Nicole Rogus-Pulia, Bryan Bednarz

**Affiliations:** 1 Department of Medical Physics, University of Wisconsin—Madison, Madison, WI, United States of America; 2 Department of Communication Sciences & Disorders, University of Wisconsin-Madison, Madison, WI, United States of America; 3 Geriatric Research Education and Clinical Centers, William S. Middleton Memorial Veterans Hospital, Madison, WI, United States of America; 4 Departamento de Ciencias de la Rehabilitación en Salud, Universidad del Bío-Bío, Chillán, Chile; 5 Department of Surgery, University of Wisconsin—Madison, Madison, WI, United States of America; 6 Department of Medicine, University of Wisconsin—Madison, Madison, WI, United States of America

**Keywords:** deep learning, Dysphagia, image analysis

## Abstract

Anatomical segmentations generated using artificial intelligence (AI) have the potential to significantly improve video fluoroscopic swallow study (VFS) analysis. AI segments allow for various metrics to be determined without additional time constraints streamlining and creating new opportunities for analysis. While the opportunity is vast, it is important to understand the challenges and limitations of the underlying AI task. This work evaluates a bolus segmentation network. The first swallow of thin or liquid bolus from 80 unique patients were manually contoured from bolus first seen in the oral cavity to end of swallow motion. The data was split into a 75/25 training and validation set and a 4-fold cross validation was done. A U—Net architecture along with variations were tested with the dice coefficient as the loss function and overall performance metric. The average validation set resulted in a dice coefficient of 0.67. Additional analysis to characterize the variability of images and performance on sub intervals was conducted indicating high variability among the processes required for training the network. It was found that bolus in the oral cavity consistently degrades performance due to misclassification of teeth and unimportant residue. The dice coefficients dependence on structure size can have substantial effects on the reported value. This work shows the efficacy of bolus segmentation and identifies key areas that are detriments to the performance of the network.

## Introduction

Dysphagia is characterized by either reduced safety (i.e., invasion of food or liquid into the airway) or efficiency (accumulation of residue in the oral cavity or pharynx after the swallow) during swallowing. Dysphagia affects 8% of the world’s population, or almost 600 million people [1]. In the US, about one in 25 adults each year will experience a swallowing problem in their lifetime [2]. A report by the Agency for Health Care Policy and Research (AHCPR; now the Agency for Healthcare Research and Quality [AHRQ]) estimates that approximately one third of patients with dysphagia will develop pneumonia and that 60,000 individuals die each year in the US from such complications [3].

Successful diagnosis and management of dysphagia is highly predicated on the use of instrumentation to determine specific anatomical and physiological abnormalities. Currently, the most used procedure to diagnose dysphagia is the video fluoroscopic swallow (VFS) study. Current analysis methods rely heavily on visual interpretation of VFS studies. Human interpretation in such a manner is often time-intensive and will always contain errors in the form of noise and bias. This challenge is especially critical to swallowing as the biomechanics of this physiology occur within a 0.6 to 1 s interval over hundreds of imaging frames [4]. While increased training and standardization practice can improve these errors [5], it is well accepted that the use of computer-aided diagnostic tools is a necessity for accurate assessment and management of dysphagia and its associated complications [6, 7].

Identifying regions of interest (i.e., segmentations) plays a key role in medical image analysis. The ability to discern one region from another usually translates to a change in pixel value or signal depending on the image modality. The identification of a region allows for understanding of changes in that region’s pathology. Segmentation of VFS to identify and track structures that are important in swallowing is beginning to play an important role in dysphagia diagnosis and characterization, particularly in research settings. This may include bony structures like the hyoid and cervical vertebrae as well as fluid components like the bolus. The ability to auto-segment these structures would allow for in depth analysis and access to quantitative measures of swallowing without increasing the time dedicated to manual frame-by-frame VFS interpretation. Therefore, there is a compelling need for fast and accurate auto segmentation of VFS. This work presents the development of automated bolus segmentation in VFS using artificial intelligence methods.

## Methods

### Data acquisition and processing

Eighty unique VFS videos were retrospectively collected from a patient cohort of mixed medical diagnoses. All videos used were anonymized and encoded. Selection criteria limited the videos to the first full swallow of a thin liquid bolus. Videos were also chosen to equally represent the spectrum of dysphagia severity as determined based on Penetration-Aspiration scale ratings. Image quality was not used as a factor for selection to better represent the degree of image variability that may be present in a naturalistic setting. Frame rate varied between 15 or 30 frames per second and were recorded in the sagittal plane at a resolution of 480 × 720 pixels on a TIMS MVP Recording System^™^. All images were acquired in the setting of one hospital. Image preprocessing included cropping to 480 × 480 and removal of frames after the end of swallow related motion.

### Manual contouring

The bolus was manually segmented frame-by-frame by three expert raters using ITK-Snap. The resulting segmentations were a binary mask in which a value of 1 indicates the presence of a bolus pixel and a 0 for non-bolus pixels. Initially the boluses from two full length videos were contoured by all three raters. Videos were selected to represent high- and low-quality images. The simultaneous truth and performance level estimation (STAPLE) [8] algorithm was used to provide a probabilistic estimate of the ground truth of the bolus contour. Raters performed with an 80% or greater dice coefficient when compared to the STAPLE estimate. STAPLE results were used to inform contouring procedure and gauge the initial consistency of the raters. Prior to dataset contouring, a protocol was established to ensure consistency among raters for image viewing parameters in ITK Snap and contouring methods.

Additionally, the frames that certain bolus related timing measures occur on were recorded by two trained raters. A consensus was established between both raters to ensure reliability of the recorded frames. VFS intervals were used to gauge the performance of the network during specific bolus events. To understand the performance of the algorithm at a more granular level throughout the temporal duration of a swallow, each video was further subdivided based on 7 different temporal events significant to bolus transit. These events included: (1) Bolus First Seen in Oral Cavity (BFSOC), the first frame in which a bolus is visualized in any amount beyond the anterior lips; (2) Posterior Bolus Movement (PBM), the first frame in which the bolus passing a line joining the anterior inferior pterygoid bone and the anterior inferior cricoid cartilage; (3) Bolus Passing Mandible (BPM), the first frame in which the bolus head has passed the superior ramus of the mandible; (4) Hyoid Burst (HB), the first frame showing brisk anterior superior movement of the hyoid bone associated with swallowing; (5) Bolus Tail Passing Mandible (BTPM), the first frame in which the tail of the bolus has passed the superior ramus of the mandible; Bolus Head in the UES (BHUES), the first frame in which the head of the bolus is seen entering the upper esophageal sphincter (UES); Bolus Tail in the UES (BTUES), the first frame in which the tail of the bolus has passed the UES. These temporal events were established by two lab-trained expert raters who assessed all swallows in duplicate. All discrepancies regarding temporal events were resolved via a consensus process.

### Network architecture

A VFS video consists of frames with two spatial dimensions that are ordered sequentially in time. Therefore, a 2D U-Net was chosen to segment the bolus in each VFS video frame. A U-Net is a convolutional neural network developed originally for biomedical images by Ronneberger *et al* [9]. U-Net and its variations are used for a variety of applications including segmentation tasks for different medical imaging modalities. U-Net is a symmetric network composed of a contracting path and expanding path. The contracting path consists of blocks of two consecutive convolution layers with a max pooling layer. The contracting path downsizes the image while increasing its depth through consecutive convolutions. The expanding path utilizes transposed convolutions to effectively act as an up-sampling technique where the end size is the same as the original input. Additional connections between the contracting and expanding path can be used to incorporate information from previous layers. The output layer of the U-Net is a convolutional layer with a filter size of 1 × 1 which outputs a segmentation map of the same dimensions as the input image. Both a U-Net and a variation using a residual block in place of the contracting portion were tested.

### Training

A 2D U-Net was developed based off the original model, but adjustments were made for ease of hyperparameter tuning and model modifications. A 4-fold cross validation was done with a rough 75%/ 25% training and testing split across image series to prevent data leakage. The dataset consisted of 9285 frames with each validation fold containing around 2300 frames depending on the number of frames in each video. To evaluate the performance of the network, the dice coefficient was used. The dice coefficient is used to gauge the similarity of two samples, where in the case of a segmentation a perfect match between ground truth and prediction contour would yield a value of 1 while the opposite would yield 0.

The optimizer Adam was used since it provides added advantages over stochastic gradient descent. Both the Tversky and Dice loss were investigated. Tversky loss is similar Dice with an additional parameter to adjust penalization of false negatives [10]. Various learning rates were tested with the starting rate being 1e-4. Models were trained for 35 epochs after testing longer trainings up to 55 epochs with no significant performance gain. Image augmentations including rotations, shifts, and zoom were applied to the training images to avoid overfitting. Image augmentations were set to indicate realistic variations present in acquisitions that would occur with patient positioning and movement being reflected in rotations and others. For this reason, augmentations such as flipping were not used. Models were trained on a NVIDIA 8GB Quadro P4000 and NVIDIA DGX using Tensorflow Keras and standard Python libraries.

## Results

**Figure 1.**
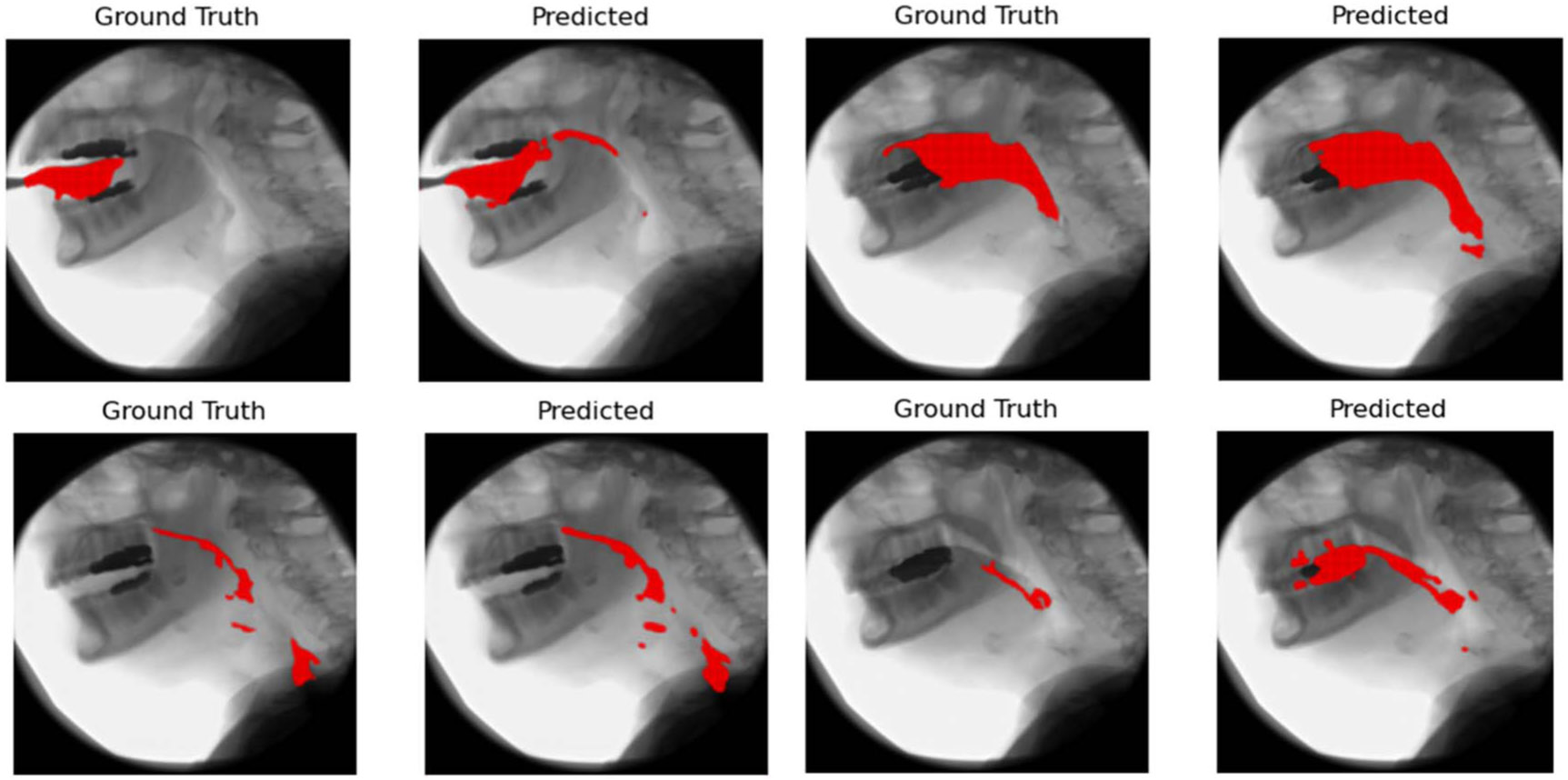
Images taken from a single video at various timepoints showing the ground truth bolus and predicted bolus.

Ground truth and predicted bolus contours at different time points are shown in figure 1. Predicted contours generally match the ground truth closely with small discrepancies at darker regions of the image or in the oral cavity.

**Figure 2.**
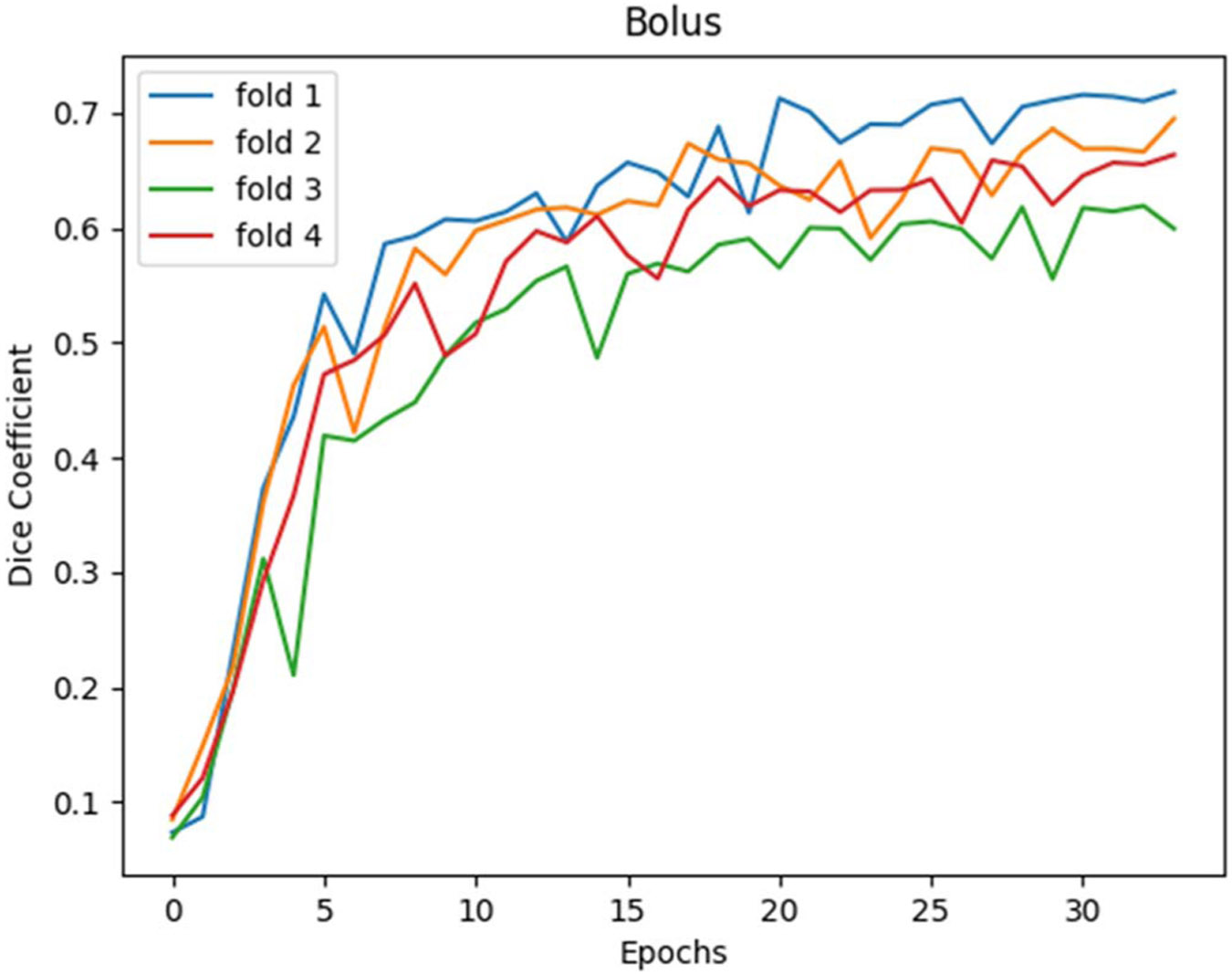
4-fold cross validation dice curves for the 25% partitions.

A U-net consisting of four levels resulted with 4-fold cross validation resulted in best performance dice coefficients of 0.62,0.65,0.69, and 0.72 for an average dice coefficient of 0.67. Figure 2 shows the testing set curves.

Network performance rapidly increased in early epochs as expected with a plateau for both training and validation as learning capacity was achieved. The additions of drop out and image augmentations were added to prevent overfitting and improve generalization of the network. All further analysis will be regarding the model validated on fold 2 of data. On an image basis, the performance of the network ranged from 0.28 to 0.81 for full image series. 78% of image series were within 1 standard deviation of the average validation dice of 0.66.

**Figure 3.**
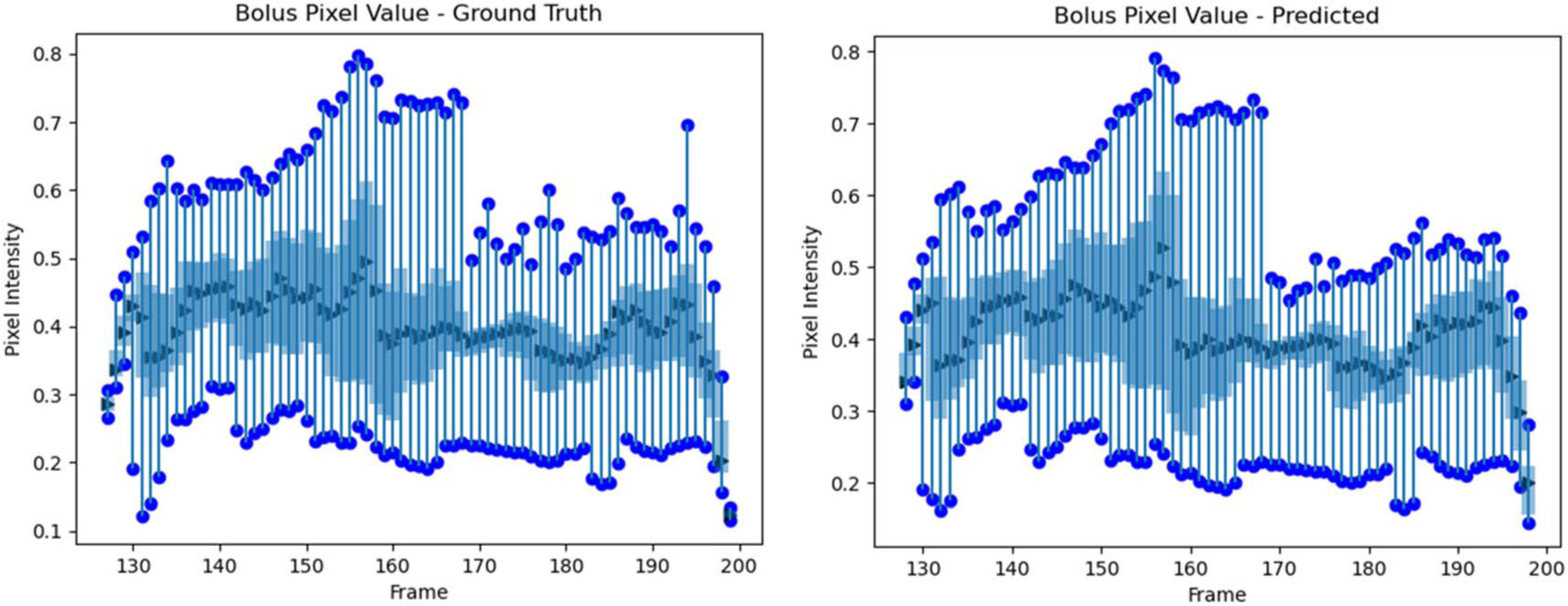
The distribution of pixel values associated with the bolus contour using both the ground truth and predicted contour on an individual frame basis. Darker arrows represent the median value while the shaded bars are 25th and 75th percentile. Minimum and maximum values are shown as points for each frame.

Examining the predicted contours on a pixel level, across 18 images, the median pixel value for the bolus was 0.23 ± 0.07 with 25th and 75th percentile values at 0.16 ± 0.06 and 0.33 ± 0.07. This ranges from the 25th to 75th percentile encompasses 17% of the inherent pixel value range of the image. Widening the percentile to 10th and 90th, the values then encompass 31% of the image range from 0.13 to 0.43. An example of the pixel distribution per frame is shown in figure 3.

The distribution of pixel values associated with the bolus varied greatly across all frames, with the larger variations spanning over 60% of the image range. Comparison of the two distributions shows general alignment in the median value of the contoured bolus and 25th to 75th percentile range. This example series did have a median value that was consistently higher than the average of the dataset. Other images showed no trend of bolus value range when compared to each other.

The Dice coefficient did not vary dramatically between each of the 15 timing intervals. The interval from when the bolus is first seen in the oral cavity to the hyoid burst resulted in a dice coefficient of 0.69. The interval of hyoid burst to bolus entering the UES resulted in a dice coefficient of 0.7. Lastly the dice coefficient for the interval from the bolus entering UES to full clearance from pharynx was 0.62. A select few cases and some intervals are shown below in the table. The results from partition 2 are shown below in table 1.

An example case where the pharynx and oral cavity were separated showed improved dice coefficients in later aspects of the swallow as shown in table 2. Reduced accuracy in early stages was primarily due to lack of bolus in the pharynx. In addition, early leakage of the bolus in the pharynx occurred during the selected swallow image which leads to bolus presence in the pharynx before the swallow.

During the initial portion of the video, the oral cavity acts as a large predictable area which leads to the higher dice coefficients for the earlier portion of the swallow as compared to cropping the oral cavity out of the image. As the swallow progresses, removing the oral cavity leads to increased performance later in the swallow as this eliminates over prediction in the oral cavity.

## Discussion

The purpose of this study was to evaluate the performance of a standard CNN on a diverse VFS dataset. The long-term goal of this work is to improve and streamline VFS analysis with AI predicted bolus contours. These contours would allow for determination of a variety of metrics relevant to the proper diagnosis and treatment of a patient’s swallowing disorder.

It is of note that the Dice coefficients presented in this work are lower than what is generally expected or accepted for other imaging modalities such as CT or MRI. There are many segmentation challenges that are unique to VFS when compared to other imaging modalities.While VFS is a 3D image, the third dimension is time and not depth. Significant changes in the shape of the bolus and its kinetics can occur over short periods of time. Additionally, the image quality seen in VFS studies is much worse than other diagnostic methods, especially those that include contrast-enhancement.

**Figure 4.**
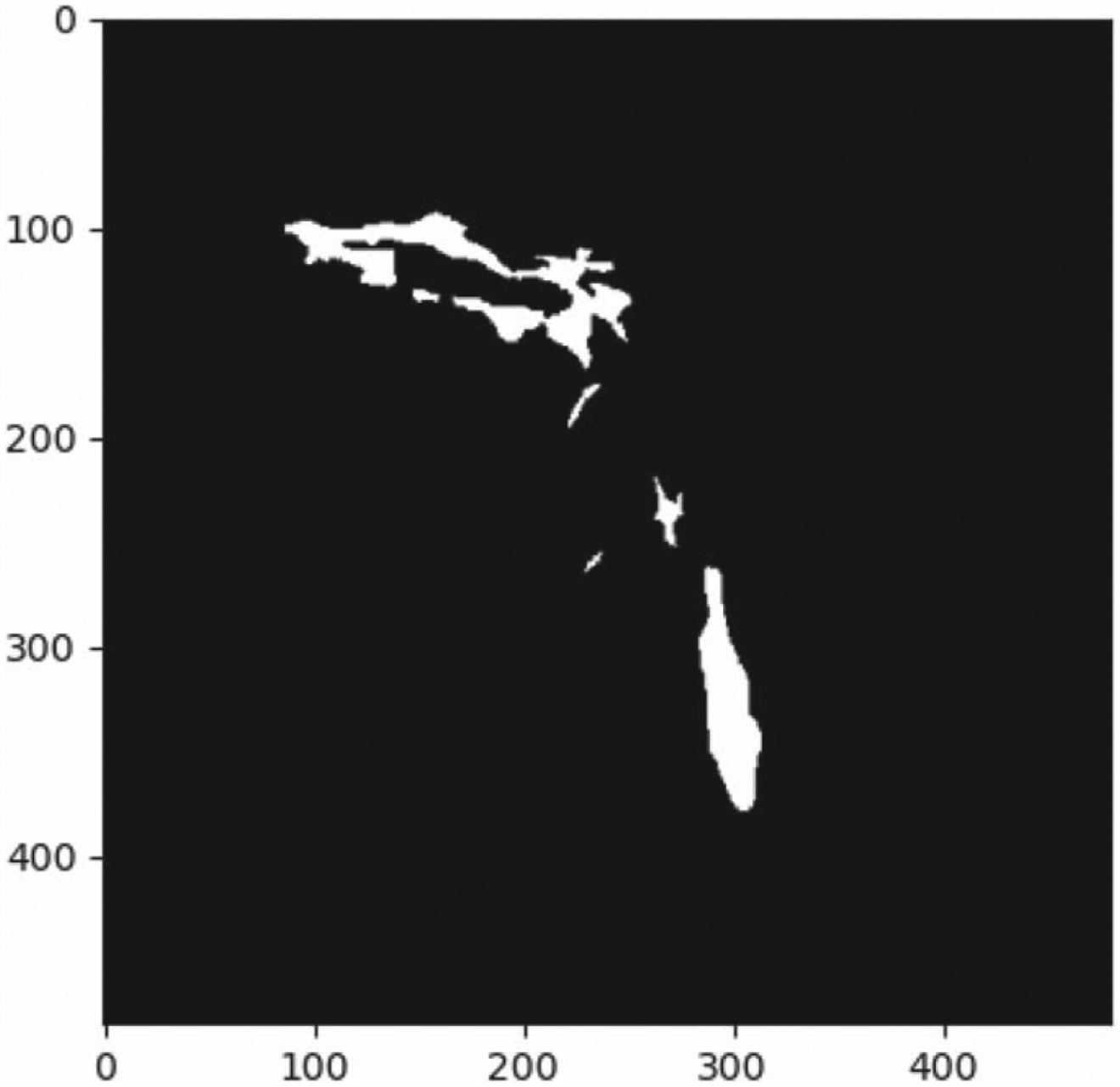
(A) single frame of the ground truth mask showing the large amount of bolus present in the oral cavity during an end stage of a swallow when compared to the important volume in the pharynx.

To the best of our knowledge, only one other group has evaluated AI-based bolus segmentation. Caliskan *et al* reported an overlap value of 0.71 compared to our reported value of 0.66 [11]. There are multiple explanations for this difference. First, the authors used a Mask-RCNN which uses a more complex architecture for segmentation compared to U-Net. Also, the authors used a greater number of swallows from fewer patients resulting in less variation across images. As shown above, there is a significant amount of patient-to-patient variation in VFS, which is likely more impactful than the type of bolus presented, or the phases of the swallow included. Finally, the authors truncated the swallow to only include the time from when the bolus entered the pharynx to full bolus clearance from the pharynx. However, our findings highlighted in figure 4 demonstrate that the teeth may be confused with the bolus so it is important to evaluate performance throughout the entirety of the swallow rather than only certain portions.

Further investigation of these results revealed that bolus contours in the oral cavity were highly variable given that the bolus was inaccurately identified as teeth or other oral structures such as dentures or fillings, as these objects have a similar pixel value to that of the bolus. This uncaptured portion of the bolus residual is primarily responsible for lowering the overall performance. For example, a qualitative assessment of the image series that resulted in a 0.28 overall dice coefficient demonstrated that the image series itself was of high enough quality to discern the bolus from surrounding tissue. There was also no extreme movement or artifact that would lead to such low performance. Throughout the entirety of the swallow, there was either bolus or residue present in the oral cavity as seen in figure 4. We should note that the effects of missing large amounts of oral cavity residue on the overall dice coefficient also reflect the shortcomings of the dice coefficient metric. The dice coefficient yields the absolute similarity between two masks with no weighting to shape, size, or location of pixels. This is a well-known limit given that, for small structures, minor differences in similarity result in a larger change in the coefficient. In addition to missed residue in the oral cavity, other image series that underperformed were generally due to worse contrast or large amounts of patient motion.

While some variation in network performance is noted among temporal intervals, it appears that the most significant barriers to network performance do not appear to be interval specific but rather video specific. Four videos in the validation set across intervals and on average had poor predicted contours. When excluding these underperformers, the overall dice coefficient increases to 0.70. A visual inspection of these videos would not place them as low or worse contrast when compared to the rest of the dataset. A VFS has a pixel range of 0 to 1 where 0 represented as dark space indicates a low incidence of photons onto the detector while the opposite is true for pixels with a value of 1. This scales linearly from 0 to 1 on the image. It is expected that higher attenuating structures such as bone would be represented as darker pixels. Compared to an imaging modality like CT, VFS has a smaller pixel range from 0 to 1 that is based on normalized intensity compared to CT where Hounsfield units are used. Hounsfield units are based on arbitrary assigned density values of air and water although provide a reference for other structures. This lack of reference for VFS leads to large variation in pixel intensity for structures that should be similar.

As shown in figure 3, 30% of the image range is captured solely within the bolus contour. While this is arguably a large range of values for a single contour to span, pixel value is one component that a neural net uses to learn. The ability of a neural network to localize a particular feature alleviates the confounding values from similarly attenuating structures such as bone. While most bolus pixels fall into a 17% range, this does raise questions about the accuracy of labeling a pixel based solely on visual inspection and contrast to neighboring pixels. This variation in pixel value generally occurs with edge pixels and trace amounts of bolus as seen with residue. This limitation is of particular concern, as detection of bolus edges is crucial to measures of swallow timing, safety, and efficiency. Given that the primary mode of discerning pixels is visual, the wide range of possible bolus values indicate that additional methods or thresholds may be required for manual contouring.

The use of denoising algorithms represents a possible solution that could improve the quantitative analysis presented here as well as the clarity of visualization of structures during the initial contouring process. VFS analysis is heavily dependent on identification of both edge and small pixels which can be lost with some denoising processes. Before utilizing a denoising process, it would be beneficial to determine the effects of such processes on diagnostic endpoints for VFS.

## Conclusions

This study was able to develop a segmentation network capable of achieving an accuracy of 0.66 with a large range of image quality and patient series using a standard U-Net. Through a variety of testing additions to the U-Net in the form of residual blocks did not offer a significant improvement while increasing the number of trainable parameters. It seems that as opposed to higher complexity networks that the challenge still lies with the data itself. The dataset for VFS is very challenging. There is not only variation of the underlying condition represented in different PAS scores, but also variation with image acquisition parameters and quality such as FOV and contrast. On top of this the clinical element further complicates this as patient positioning and motion add further variables. For these reasons, we believe that a much larger data set is required in order to achieve a well performing generalized algorithm.

Regarding future implications of this work, while more research is currently needed to optimize the auto segmentation process, these preliminary results are promising and hold positive implications for future assessment and management of dysphagia. It is now well-accepted within the field that use of instrumentation is crucial to accurate diagnosis and treatment of swallowing disorders [6, 12, 13]. However, as our understanding of the true complexity of swallowing physiology continues to advance, it is important to note that the physiology of swallowing is not beholden to our perceptual capacities. In other words, we may discover that truly accurate understanding of this mechanism may be beyond the capabilities of perceptual assessment of instrumental findings thereby requiring further technological supplementation. Furthermore, just as other fields of healthcare have already benefited from the implementation of AI, [5, 14-16] it can be expected that the field of dysphagia stands to benefit as well. The authors would like to emphasize that this implementation should be viewed as an opportunity for advancement of the field for clinicians, as opposed to a loss of clinical responsibility or autonomy. While AI will serve to bolster the understanding of the swallowing mechanism and streamline the assessment process, a competent clinician is still needed to translate findings to management and to balance potential treatment plans with the needs and desires of the patient.

## Figures and Tables

**Table 1.** The Dice coefficient for swallow intervals. Empty cells indicate the end interval event occurred prior to the starting event.

ID	BFSOC:BPM	BFSOC:BTPM	BPM:HB	BPM:BTUES	BTPM:BTUES
0	0.08	0.33	0.53	0.51	0.44
1	0.79	0.79	0.80	0.72	0.65
2	0.70	0.74		0.82	0.80
3	0.59	0.65		0.63	0.45
4	0.74	0.79		0.82	0.76
5	0.49	0.48	0.42	0.35	
6	0.76	0.76			
7	0.86	0.851	0.89	0.81	0.71
8	0.46	0.66	0.69	0.69	0.63
9	0.75	0.73	0.63	0.59	0.55
10	0.51	0.46	0.43	0.451	0.48
11	0.69	0.83	0.79	0.80	0.70
12	0.62	0.73		0.75	0.71
13	0.76	0.62		0.62	0.66
14	0.78	0.55	0.55	0.53	0.11
15	0.80	0.82	0.86	0.85	0.80
16	0.86	0.86	0.81	0.80	0.69
17	0.67	0.71	0.84	0.73	0.54
Average	0.66	0.69	0.69	0.68	0.60

**Table 2.** An example case where analysis is repeated with the removal of the bolus from oral cavity compared to the full image.

Oral cavity	BFSOC:BPM	BFSOC:BTPM	BPM:HB	BPM:BTUES	BTPM:BTUES
Kept	0.80	0.79	0.80	0.72	0.64
Removed	0.41	0.67	0.61	0.78	0.74

## Data Availability

The data cannot be made publicly available and is not available upon request. The data cannot be made publicly available upon publication due to legal restrictions preventing unrestricted public distribution.
